# Effect of supplementation with different sources and levels of zinc, copper, and manganese on the reproductive development of laying hens

**DOI:** 10.1016/j.psj.2025.105306

**Published:** 2025-05-16

**Authors:** Joelma Vasconcelos Celestino da Silva, Adiel Vieira de Lima, Anna Neusa Eduarda Ferreira de Brito, Thamires da Silva Ferreira, Isabelle Naemi Kaneko, Leonardo Augusto Fonseca Pascoal, Matheus Ramalho de Lima, Ricardo Romão Guerra, Apolônio Gomes Ribeiro, Jorge Cunha Lima Muniz, Lucas Rannier Ribeiro Antonino Carvalho, Fernando Guilherme Perazzo Costa

**Affiliations:** a Animal Science Department, Universidade Federal da Paraíba, Areia, PB, Brazil; b Animal Science Department, Universidade Federal de Rondônia, Presidente Medici, RO, Brazil; c Animal Science Department, Universidade Federal da Paraíba, Bananerias, PB, Brazil; d Animal Science Department, Universidade Federal Rural do Semi-Árido, Mossoró, RN, Brazil; e Animal Science Department, Universidade Federal de Viçosa, Viçosa, MG, Brazil; f Department of Physiology and Pharmacology Karolinska Institutet, Stockholm Sweden Biomedicum, 5B, Solnavägen 9, Stockholm S-171 77, Sweden

**Keywords:** Poultry, Physiology, Morphology, Nutrition, Oviduct

## Abstract

The study evaluated the effects of zinc (Zn), copper (Cu), and manganese (Mn) supplementation from different sources and levels on the performance and reproductive development of Hy-Line W-80 laying hens. A total of 364 birds were assigned to a completely randomized 2 × 2 factorial design, with two mineral sources (sulfate vs. mineral hydroxy‑analogue of methionine) and two supplementation levels (recommended vs. higher than recommended), totaling four treatments with seven replicates. Birds were fed the experimental diets from hatch to 54 weeks of age (378 days). Performance parameters included body weight, feed intake, feed conversion, age at first egg, first egg weight, and egg production from 17 to 54 weeks. Morphological aspects of the magnum and uterus (height, width, and number of folds) were assessed at pre-laying, pre-peak, and laying phases by slaughtering one bird per replicate at 17, 23, and 54 weeks. The first egg was laid at 119 days (34.79 g) in birds receiving sulfate with recommended mineral levels (FS + NR). The shortest interval between first eggs occurred in birds supplemented with the hydroxy‑analogue at higher levels (FQ + NS), which also resulted in improved egg number, weight, and production from 20 to 54 weeks. FS + NR led to better microscopic development of the magnum and uterus at 54 weeks. Overall, Zn, Cu, and Mn supplementation from hatch using the sulfate source at recommended levels benefited initial performance and reproductive development of laying hens until 54 weeks of age.

## Introduction

The improvement in egg production per housed hen is one of the main selection criteria in laying hen breeding. In addition to genetics, factors such as housing systems and nutrition directly influence bone strength and eggshell quality (Pereira et al., 2020). Minerals are key elements in the nutrition of laying hens, playing essential roles in various metabolic processes throughout their lives. From the early days, minerals contribute to skeletal system development, sexual maturity, and egg formation (Elnesr et al., 2024). However, nutritional requirements for minerals are mainly studied during the productive phase due to their importance in eggshell formation. Few studies have addressed the role of trace minerals in laying hens before egg production and their potential beneficial interactions with the endocrine and reproductive systems before reproductive age.

The primary goal of the pre-laying phase is to develop uniform flocks with an optimal body weight, as this variable directly influences sexual maturity, as demonstrated in previous studies (Leeson and Summers, 2001; Renema et al., 2007; Albino et al., 2014; D’Agostini et al., 2017). Sexual maturity is marked by significant morphological changes in the ovary and the development of the oviduct (infundibulum, magnum, isthmus, uterus, and vagina) (Poultry Hub Australia, 2025). The first egg can be laid as early as 16 weeks of age, depending on the genetic lineage.

During this period, metabolic activity increases rapidly, raising the birds' nutrient requirements. Understanding the potential effects of trace minerals during growth and sexual maturity development can support more precise nutritional decisions, improving productivity during the laying phase. The trace minerals zinc (Zn), copper (Cu), and manganese (Mn) are particularly relevant in laying hen diets as they play active roles in egg and eggshell composition (Pereira et al., 2020).

Zinc is associated with reproductive system functions, bone formation, and serves as a cofactor for carbonic anhydrase, an enzyme essential for eggshell formation (Leeson and Summers, 2001; Silva and Pascoal, 2014). Copper contributes to cartilage formation, eggshell membrane integrity, and pigmentation (Scheideler, 2008). Additionally, it has antimicrobial properties and supports intestinal health (Wang et al., 2014). Manganese is involved in the development and function of the reproductive system (Underwood and Suttle, 1999; Swiatkiewicz and Koreleski, 2008).

Mineral supplementation can be achieved through inorganic sources (oxides, sulfates, chlorides, carbonates, or phosphates) (Silva and Pascoal, 2014) or organic sources, in which a metal ion is bound to an organic molecule (McDowell, 2003). Depending on the type of ligand, these can form metal complexes (complexes, chelates, or proteinates) (Rutz and Murphy, 2009), which provide more stable structures in the organism compared to inorganic sources (Richards et al., 2010).

Given these considerations, there is a need to better understand how the sources and levels of trace minerals provided during the early growth phase affect the subsequent reproductive development of lightweight laying hens. Although the role of zinc, copper, and manganese in egg formation is well established, limited information is available regarding their influence during the pre-laying period and their long-term effects on oviduct morphology and egg production. It is hypothesized that supplementation with more bioavailable sources, such as hydroxy‑methionine analogs, and higher inclusion levels of these minerals will promote better early performance, reproductive organ development, and egg production compared to inorganic sulfate sources at recommended levels. Therefore, this study aimed to evaluate the comparative effects of sulfate and hydroxy‑methionine analog sources of zinc, copper, and manganese at different inclusion levels on the early performance of birds (1–15 weeks of age) and their reproductive system (17–54 weeks of age) by assessing egg production and the morphology of the magnum and uterus in lightweight laying hens.

## Material and methods

The experimental protocol was approved by the Animal Ethics Committee (CEUA) of the Federal University of Paraíba, Brazil, under approval certificate N^o^. 95620909-19.

### Experimental design and facilities

The experiment was conducted at the poultry sector of the Department of Animal Science, Center for Agricultural Sciences, Federal University of Paraíba, Campus II, located in the municipality of Areia, Paraíba, Brazil. Environmental conditions, including temperature and relative humidity, were recorded daily throughout the experiment, with average values of 23.8°C and 82 % RH.

A total of 364 Hy-Line W-80 laying hens were initially used, starting from one day of age, and selected based on an average body weight of 45 g. Throughout the study, due to the collection of biological material for histological analysis, one bird per experimental unit was slaughtered. The slaughtering criterion was based on changes in nutritional requirements as described in the lineage manual. This approach ensured that if any dietary effects occurred, they could be identified at an approximate time point. Consequently, the number of birds per treatment varied as follows:

• 364 birds in the brooding phase: pre-starter (1 to 3 weeks) (*n* = 13);
• 336 birds in the brooding phase: starter (4 to 6 weeks) (*n* = 12);
• 308 birds in the rearing phase: growth (7 to 12 weeks) (*n* = 11);
• 280 birds in the rearing phase: development (13 to 15 weeks) (*n* = 10);
• 252 birds in the pre-laying phase (16 to 17 weeks) (*n* = 9);
• 224 hens in the pre-peak phase (18 to 23 weeks) (*n* = 8);
• 196 hens in the laying phase (24 to 54 weeks) (*n* = 7).

The experimental period was divided into four phases: brooding and rearing phase (1 day to the 15th week of age), pre-lay phase (16th to 17th week of age), pre-peak laying phase (18th to 23rd week of age), and laying phase (24th to 54th week of age), resulting in 378 days of experimentation (1 day to 54 weeks of age). Four treatments were used, with seven replicates and “pre-starter (364 birds); starter (336 birds); growth (308 birds); development (280 birds); pre-laying phase (252 birds); pre-peak phase (224 birds); laying phase (196 birds)” birds per experimental unit, distributing the respective experimental units according to a completely randomized design in a 2 × 2 factorial scheme to compare different sources (sulfate and hydroxy‑methionine analog mineral) with different levels of zinc, copper, and manganese supplementation in the birds’ diet. The experimental unit was represented by the cage.

As an inorganic source, zinc sulfate (35 % Zn), copper sulfate (34.5 % Cu), and manganese sulfate (26 % Mn) were used. The organic source used was zinc chelated to hydroxy‑methionine analog (16 % Zn), copper chelated to hydroxy‑methionine analog (16 % Cu), and manganese chelated to hydroxy‑methionine analog (13 % Mn). The established levels were determined based on the minimum nutritional requirement of the birds using the inorganic source, according to the recommendations of the strain manual (Hy-Line, 2016). Thus, the amount of supplemented zinc (Zn), copper (Cu), and manganese (Mn) was 32/08/32 ppm/kg of feed, respectively, representing the recommended level (NR), and 64/16/64 ppm/kg representing the level above the recommended (NS).

Therefore, the treatments were structured as follows: Treatment 01 = Sulfate source + recommended level (FS+NR); Treatment 02 = Sulfate source + level above recommended (FS+NS); Treatment 03 = Chelated source + recommended level (FQ+NR); Treatment 04 = Chelated source + level above recommended (FQ+NS) for Zn, Cu, and Mn, with these nomenclatures being used throughout the text.

Two poultry houses were used in this study. The brooding and rearing house was built of masonry with clay tile roofing, metal cages coated with plastic material, and a plastic-mesh floor with dimensions of 31 × 57 × 77 cm (depth × length × width). Each cage initially housed 13 birds, providing a stocking density of approximately 337.6 cm² per bird. All birds were provided with infant drinkers and feeders, which were later replaced with PVC trough feeders and nipple drinkers before beak trimming (at 10 days of age) to allow the chicks to adapt. The birds had ad libitum access to water and feed. Heating was provided by incandescent lamps until the 15th day of age.

The laying house was a conventional laying facility, covered with clay tiles, with trough-type feeders and nipple drinkers, where the birds were housed in galvanized wire cages measuring 24 × 37 × 41 cm (depth x length x width), with ad libitum access to water and feed. In the first house, the birds were housed from day 1 until the end of the 11th week of life, when they were transferred to the laying house, where they remained from the 12th to the 54th week of age.

The lighting program recommended by the Hy-Line W-80 (Hy-Line, 2016) management guide was adopted, ensuring the absence of photoperiod effects on the birds’ sexual maturity. The lighting regime during the brooding and rearing phases started with 20 h of light and was gradually reduced to 12 h of light (natural + artificial) to prevent early sexual maturity and promote good weight gain and uniformity. The pre-lay phase started with 12 h of light and ended with 13 h of light (natural + artificial). The pre-peak and laying phases experienced gradual increases of 15 min per week until reaching 16 h of light.

Table 1 details the lighting program adopted throughout the experiment, consisting of 12 h of natural light, with artificial lighting provided continuously by fluorescent lamps. Throughout the experimental period, the birds were managed under the same conditions.

**Table 1:** Detailed lighting program used in the study and air temperature and humidity values during the experimental period.

Phase	Bird Age (weeks)	Lighting (hours of light)	Temperature (°C) Avg. (max./min.)	Humidity (%) Avg. (max./min.)
Brooding: Pre-starter	1 to 3	20 to 19	25.1 (28.8 - 21.4)	83 (95 – 72)
Brooding: Starter	4 to 6	18 to 16	25.5 (29.2 - 21.8)	81 (95 – 74)
Growing	7 to 12	15 to 12	24.4 (27.7 - 21.2)	84 (97 – 69)
Developing	13 to 15	12	23.7 (26.4 - 20.9)	87 (97 – 73)
Pre-lay	16 to 17	12	22.6 (25.4 - 19.9)	88 (97 – 80)
Pre-peak	18 to 23	13 to 14:25	21.2 (23.5 - 18.8)	89 (98 – 74)
Laying	24 to 26	14:30 to 15	21.9 (25.6 - 18.2)	81 (91 – 73)
	26 to 29	15:25 to 16	21.9 (25.3 - 18.6)	84 (97 – 71)
	30 to 33	16	23.0 (27.0 - 19.1)	78 (86 – 73)
	34 to 37	16	23.9 (27.8 - 20.0)	80 (94 – 73)
	38 to 41	16	24.9 (29.3 - 20.5)	74 (83 – 64)
	42 to 45	16	25.4 (29.9 - 21.0)	75 (86 – 65)
	46 to 49	16	25.1 (29.4 - 20.9)	78 (97 – 69)
	50 to 54	16	25.1 (29.4 - 20.9)	83 (97 – 73)

### Diets

The diets were formulated using corn and soybean meal and followed the nutritional recommendations of the Hy-Line W-80 Management Guide (Hy-Line, 2016) for all ages. The calcium and methionine content present in corn, soybean meal, and the chelated source were considered during formulation and deducted when calculating the requirement for the methionine source (MHA-Ca).

Table 2 summarizes the amounts of zinc, copper, and manganese used in the different treatments in the experimental diets to achieve the required levels of Zn, Cu, and Mn: 85/15/90 ppm from the brooding phase to pre-lay and 80/8/90 ppm from the pre-peak to the laying phase, according to the Management Guide (Hy-Line, 2016).

**Table 2:** Nutritional values of zinc, copper, and manganese in the treatments studied during the 54-week experimental period in lightweight laying hens.

Treatments	1 to 3 weeks (Zn/Cu/Mn) ppm	4 to 6 weeks (Zn/Cu/Mn) ppm	7 to 12 weeks (Zn/Cu/Mn) ppm	13 to 15 weeks (Zn/Cu/Mn) ppm	16 to 17 weeks (Zn/Cu/Mn) ppm	18 to 37 weeks (Zn/Cu/Mn) ppm	38 to 54 weeks (Zn/Cu/Mn) ppm
	Brooding		Rearing		Pre-lay	Pre-peak	Laying
T1	61/ 15 /50	60/ 14 / 49	58/ 13 / 43	58/ 13 / 43	58/ 13 / 47	57/ 13 / 47	55/ 12 / 41
T2	93/ 23 / 82	92/ 22 / 81	90/ 21 / 75	90/ 21 / 75	90/ 21 /79	89/ 21 /79	87 / 21 / 75
T3	61/ 15 /50	60/ 14 / 49	58/ 13 / 43	58/ 13 / 43	58/ 13 / 47	57/ 13 / 47	55/ 12 / 41
T4	93/ 23 / 82	92/ 22 / 81	90/ 21 / 75	90/ 21 / 75	90/ 21 /79	89/ 21 /79	87 / 21 / 75

Table 3, Table 4 present the experimental diets for the brooding, growing, pre-lay, pre-peak, and laying phases.

**Table 3:** Percent composition and calculated nutritional values of experimental diets for lightweight laying hens in the rearing, growing, and pre-laying phases.

Ingredients (%)	Phases																			
	1 to 3 weeks				4 to 6 weeks				7 to 12 weeks				13 to 15 weeks				16 to 17 weeks			
	Treatments				Treatments				Treatments				Treatments				Treatments			
	T1	T2	T3	T4	T1	T2	T3	T4	T1	T2	T3	T4	T1	T2	T3	T4	T1	T2	T3	T4
Corn, 7.88 %	63.24	63.24	63.24	63.24	68.1	68.1	68.1	68.1	71.18	71.18	71.18	71.18	71.97	71.97	71.97	71.97	67.64	67.64	67.64	67.64
Soybean meal, 45.22 %	31.16	31.16	31.16	31.16	27.14	27.14	27.14	27.14	23.42	23.42	23.42	23.42	22.71	22.71	22.71	22.71	23.33	23.33	23.33	23.33
Soybean oil	1.62	1.62	1.62	1.62	0.78	0.78	0.78	0.78									0.89	0.89	0.89	0.89
Dicalcium phosphate, 18.5 %	1.8	1.8	1.8	1.8	1.78	1.78	1.78	1.78	1.35	1.35	1.35	1.35	1.4	1.4	1.4	1.4	1.45	1.45	1.45	1.45
Common salt	0.48	0.48	0.48	0.48	0.46	0.46	0.46	0.46	0.41	0.41	0.41	0.41	0.41	0.41	0.41	0.41	0.41	0.41	0.41	0.41
L-Lysine HCl	0.13	0.13	0.13	0.13	0.15	0.15	0.15	0.15	0.13	0.13	0.13	0.13					0.02	0.02	0.02	0.02
Choline chloride, 60 %	0.07	0.07	0.07	0.07	0.07	0.07	0.07	0.07	0.07	0.07	0.07	0.07	0.07	0.07	0.07	0.07	0.07	0.07	0.07	0.07
Vitamin + Se Premix¹	0.1	0.1	0.1	0.1	0.1	0.1	0.1	0.1	0.1	0.1	0.1	0.1	0.1	0.1	0.1	0.1	0.1	0.1	0.1	0.1
Potassium iodate	0.0002	0	0.0002	0.0002	0	0	0	0	0.0002	0.0002	0.0002	0.0002	0.0002	0.0002	0.0002	0.0002	0.0002	0.0002	0.0002	0.0002
Iron sulfate, 20 %	0.01	0.01	0.01	0.01	0.01	0.01	0.01	0.01	0.01	0.01	0.01	0.01	0.01	0.01	0.01	0.01	0.01	0.01	0.01	0.01
Antioxidant²	0.01	0.01	0.01	0.01	0.01	0.01	0.01	0.01	0.01	0.01	0.01	0.01	0.01	0.01	0.01	0.01	0.01	0.01	0.01	0.01
Phytase³	0.01	0.01	0.01	0.01	0.01	0.01	0.01	0.01	0.01	0.01	0.01	0.01	0.01	0.01	0.01	0.01	0.01	0.01	0.01	0.01
Limestone, 37 %	0.85	0.85	0.86	0.88	0.89	0.89	0.9	0.92	1.25	1.25	1.26	1.28	1.28	1.28	1.3	1.31	5.63	5.63	5.65	5.66
MHA-Ca⁴	0.29	0.29	0.25	0.2	0.28	0.28	0.24	0.19	0.25	0.25	0.2	0.16	0.12	0.12	0.08	0.03	0.21	0.21	0.17	0.19
Zinc sulfate, 35 %	0.009	0.018	-	-	0.009	0.018	-	-	0.009	0.018	-	-	0.009	0.018	-	-	0.009	0.018	-	-
Copper sulfate, 34.5 %	0.002	0.005	-	-	0.002	0.005	-	-	0.002	0.005	-	-	0.002	0.005	-	-	0.002	0.005	-	-
Manganese sulfate, 26 %	0.012	0.025	-	-	0.012	0.025	-	-	0.012	0.025	-	-	0.012	0.025	-	-	0.012	0.025	-	-
Zn-Met, 16 %⁵	-	-	0.02	0.04	-	-	0.02	0.04	-	-	0.02	0.04	-	-	0.02	0.04	-	-	0.02	0.04
Cu-Met, 15 %⁶	-	-	0.005	0.011	-	-	0.005	0.011	-	-	0.005	0.011	-	-	0.005	0.011	-	-	0.005	0.011
Mn-Met, 13 %⁷	-	-	0.025	0.049	-	-	0.025	0.049	-	-	0.025	0.049	-	-	0.025	0.049	-	-	0.025	0.049
Inert⁸	0.2	0.18	0.21	0.19	0.2	0.18	0.21	0.19	1.79	1.76	1.8	1.78	1.91	1.91	1.89	1.87	0.2	0.18	0.2	0.12
TOTAL	100	100	100	100	100	100	100	100	100	100	100	100	100	100	100	100	100	100	100	100
*Chemical composition*																				
ME, kcal/kg	3000	3000	3000	3000	3000	3000	3000	3000	2950	2950	2950	2950	2950	2950	2950	2950	2900	2900	2900	2900
CP, %	19.3	19.3	19.3	19.3	17.9	17.9	17.9	17.9	16.5	16.5	16.5	16.5	16	16	16	16	16	16	16	16
Digestible Met, %	0.51	0.51	0.51	0.51	0.48	0.48	0.48	0.48	0.44	0.44	0.44	0.44	0.33	0.33	0.33	0.33	0.41	0.41	0.41	0.41
Digestible Met + Cys, %	0.78	0.78	0.78	0.78	0.74	0.74	0.74	0.74	0.68	0.68	0.68	0.68	0.57	0.57	0.57	0.57	0.64	0.64	0.64	0.64
Digestible Lys, %	1.02	1.02	1.02	1.02	0.94	0.94	0.94	0.94	0.84	0.84	0.84	0.84	0.72	0.72	0.72	0.72	0.74	0.74	0.74	0.74
Calcium, %	1	1	1	1	1	1	1	1	1	1	1	1	1	1	1	1	2.7	2.7	2.7	2.7
Available Phosphorus, %	0.5	0.5	0.5	0.5	0.49	0.49	0.49	0.49	0.47	0.47	0.47	0.47	0.47	0.47	0.47	0.47	0.48	0.48	0.48	0.48
Sodium, %	0.21	0.21	0.21	0.21	0.2	0.2	0.2	0.2	0.18	0.18	0.18	0.18	0.18	0.18	0.18	0.18	0.18	0.18	0.18	0.18
Chlorine, %	0.34	0.34	0.34	0.34	0.32	0.32	0.32	0.32	0.29	0.29	0.29	0.29	0.29	0.29	0.29	0.29	0.29	0.29	0.29	0.29
Potassium, %	0.75	0.75	0.75	0.75	0.69	0.69	0.69	0.69	0.63	0.63	0.63	0.63	0.62	0.62	0.62	0.62	0.62	0.62	0.62	0.62
Zinc, ppm/kg	32	64	32	64	32	64	32	64	32	64	32	64	32	64	32	64	32	64	32	64
Copper, ppm/kg	8	16	8	16	8	16	8	16	8	16	8	16	8	16	8	16	8	16	8	16
Manganese, ppm/kg	32	64	32	64	32	64	32	64	32	64	32	64	32	64	32	64	32	64	32	64

¹Premix per kilogram of feed – Folic Acid: 30 g; Pantothenic Acid: 4.8 g; Biotin: 60 g; Niacin: 15 g; Sodium Selenite: 40 g; Vitamin A: 7,000,000 IU; Vitamin B1: 30 g; Vitamin B12: 0.00001 g; Vitamin B2: 4.5 g; Vitamin B6: 30 g; Vitamin D3: 3,000,000 IU; Vitamin E: 15,000 IU; Vitamin K: 90 g; B.H.T: 2 g; ²Ethoxyquin; ³Sunphase 5000 FYT. Phytase derived from *Escherichia coli* bactéria; ⁴MHA-Ca (methionine hydroxy-analog calcium salt): dry matter 99 %, methionine activity 84 %, protein value 49.3 %, organic calcium 12 %, ME 4,041 kcal/kg; ⁵Zn-Met: methionine activity 80 %, protein value 47 %, ME 3,823 kcal/kg; ⁶Cu-Met: methionine activity 78 %, protein value 45.8 %, ME 3,727 kcal/kg; ⁷Mn-Met: methionine activity 76 %, protein value 44.7 %, ME 3,632 kcal/kg; ⁸Washed sand.

**Table 4:** Percentual composition and calculated nutritional values of experimental diets for lightweight laying hens in the pre-peak and laying phases.

Ingredients (%)	Fases											
	18 to 37 weeks				38 to 48 weeks				49 to 54 weeks			
	Treatments				Treatments				Treatments			
	T1	T2	T3	T4	T1	T2	T3	T4	T1	T2	T3	T4
Corn, 7.88 %	63.27	63.27	63.27	63.27	67.18	67.18	67.18	67.18	64.97	64.97	64.97	64.97
Soybean meal, 45.22 %	22.22	22.22	22.22	22.22	19.3	19.3	19.3	19.3	20.3	20.3	20.3	20.3
Soybean oil	2.14	2.14	2.14	2.14	1.57	1.57	1.57	1.57	2.3	2.3	2.3	2.3
Dicalcium phosphate, 18.5 %	1.59	1.59	1.59	1.59	1.33	1.33	1.33	1.33	1.7	1.7	1.7	1.7
Common salt	0.39	0.39	0.39	0.39	0.33	0.33	0.33	0.33	0.39	0.39	0.39	0.39
L-Lysine HCl	0.23	0.23	0.23	0.23	0.14	0.14	0.14	0.14	0.09	0.09	0.09	0.09
Choline chloride, 60 %	0.07	0.07	0.07	0.07	0.07	0.07	0.07	0.07	0.07	0.07	0.07	0.07
Vitamin + Se Premix¹	0.1	0.1	0.1	0.1	0.1	0.1	0.1	0.1	0.07	0.07	0.07	0.07
Potassium iodate	0	0	0	0	0	0	0	0	0.1	0.1	0.1	0.1
Iron sulfate, 20 %	0.01	0.01	0.01	0.01	0.01	0.01	0.01	0.01	0	0	0	0
Antioxidant²	0.01	0.01	0.01	0.01	0.01	0.01	0.01	0.01	0.01	0.01	0.01	0.01
Phytase³	0.01	0.01	0.01	0.01	0.01	0.01	0.01	0.01	0.01	0.01	0.01	0.01
Coarse limestone, 37 %	4.58	4.58	4.58	4.59	5.18	5.18	5.18	5.18	7.28	7.28	7.28	7.28
Fine limestone, 37 %	4.58	4.58	4.58	4.59	4.24	4.24	4.26	4.27	2.33	2.33	2.33	2.33
MHA-Ca⁴	0.4	0.4	0.35	0.31	0.26	0.26	0.21	0.17	0.2	0.2	0.16	0.12
Zinc sulfate, 35 %	0.01	0.02	-	-	0.01	0.02	-	-	0.01	0.02	-	-
Copper sulfate, 34.5 %	0	0	-	-	0	0	-	-	0	0	-	-
Manganese sulfate, 26 %	0.01	0.02	-	-	0.01	0.02	-	-	0.01	0.02	-	-
Zn-Met, 16 %⁵	-	-	0.02	0.04	-	-	0.02	0.04	-	-	0.02	0.04
Cu-Met, 15 %⁶	-	-	0.01	0.01	-	-	0.01	0.01	-	-	0.01	0.01
Mn-Met, 13 %⁷	-	-	0.02	0.05	-	-	0.02	0.05	-	-	0.02	0.05
Inert⁸	0.2	0.18	0.21	0.19	0.2	0.18	0.21	0.19	0.2	0.18	0.22	0.21
TOTAL	100	100	100	100	100	100	100	100	100	100	100	100
*Chemical composition*												
ME, kcal/kg	2860	2860	2860	2860	2860	2860	2860	2860	2890	2890	2890	2890
CP, %	15.5	15.5	15.5	15.5	14.3	14.3	14.3	14.3	14.91	14.91	14.91	14.91
Digestible Met, %	0.41	0.41	0.41	0.41	0.42	0.42	0.42	0.42	0.39	0.39	0.39	0.39
Digestible Met + Cys, %	0.77	0.77	0.77	0.77	0.63	0.63	0.63	0.63	0.6	0.6	0.6	0.6
Digestible Lys, %	0.82	0.82	0.82	0.82	0.73	0.73	0.73	0.73	0.71	0.71	0.71	0.71
Calcium, %	4.08	4.08	4.08	4.08	4.07	4.07	4.07	4.07	4.22	4.22	4.22	4.22
Available Phosphorus, %	0.5	0.5	0.5	0.5	0.44	0.44	0.44	0.44	0.4	0.4	0.4	0.4
Sodium, %	0.17	0.17	0.17	0.17	0.17	0.17	0.17	0.17	0.18	0.18	0.18	0.18
Chlorine, %	0.27	0.27	0.27	0.27	0.27	0.27	0.27	0.27	0.26	0.26	0.26	0.26
Potassium, %	0.59	0.59	0.59	0.59	0.54	0.54	0.54	0.54	0.56	0.56	0.56	0.56
Zinc, ppm/kg	32	64	32	64	32	64	32	64	32	64	32	64
Copper, ppm/kg	8	16	8	16	8	16	8	16	8	16	8	16
Manganese, ppm/kg	32	64	32	64	32	64	32	64	32	64	32	64

^1^Premix per kilogram of feed – Folic Acid: 30 g; Pantothenic Acid: 4.8 g; Biotin: 60 g; Niacin: 15 g; Sodium Selenite: 40 g; Vitamin A: 7,000,000 IU; Vitamin B1: 30 g; Vitamin B12: 0.00001 g; Vitamin B2: 4.5 g; Vitamin B6: 30 g; Vitamin D3: 3,000,000 IU; Vitamin E: 15,000 IU; Vitamin K: 90 g; B.H.T: 2 g; ²Ethoxyquin; ³Sunphase 5000 FYT. Phytase derived from *Escherichia coli* bactéria ^4^MHA-Ca (methionine hydroxy analog calcium salt): 99 % dry matter, 84 % methionine activity, 49.3 % protein value, 12 % organic calcium, ME 4,041 kcal/kg ^5^Zn-Met: 80 % methionine activity, 47 % protein value, ME 3,823 kcal/kg ^6^Cu-Met: 78 % methionine activity, 45.8 % protein value, ME 3,727 kcal/kg ^7^Mn-Met: 76 % methionine activity, 44.7 % protein value, ME 3,632 kcal/kg ^8^Washed sand.

### Performance

The initial performance variables analyzed included initial weight (IW, g), final weight (FW, g), weight gain (WG, g), feed intake (FI, g/bird/day), and feed conversion ratio (FCR, kg/bird). Measurements were determined based on changes in birds’ nutritional requirements (1-3, 4-6, 7-12, 13-15 weeks of age). IW and FW were calculated by individually weighing birds at the beginning and end of each specified period using a scale. WG was determined as the difference between final and initial body weights. FI was calculated as the difference between the total feed offered and the feed leftovers in each experimental unit. FCR was obtained by dividing WG by FI. Mortality was recorded daily to adjust FI, WG, and FCR. Corrections for initial performance parameters were made by considering the number of dead birds, feed leftovers, and the weight of remaining birds in each unit.

### Age at first egg and egg production

The first egg laid was used as a reference point for recording egg production and age at first egg. Eggs were collected twice daily (9:00 AM and 3:00 PM), and their weights were recorded in the late afternoon using an electronic scale with a precision of 0.01 g. In case of mortality, necessary corrections were applied.

The production variables analyzed included age at first egg (days), weight of first eggs (g), interval between first eggs (days), total number of eggs (units), egg weight (g), and egg production (eggs/bird/day), calculated by dividing the total number of eggs per unit by the number of birds and multiplying by 100, followed by the calculation of treatment means. To better understand birds’ responses to diets, the number of eggs, egg weight, and egg production were evaluated from 17 to 54 weeks of age. When birds reached 24 weeks, egg weight was analyzed at 28-day intervals, with all eggs weighed over the last three days of each interval, totaling six collections in this period (24 to 54 weeks).

### Morphometry of the female reproductive system

For reproductive system morphometry (magnum and uterus), birds were selected based on the average body weight of each experimental unit, and one bird per unit (*n* = 28) was euthanized at the end of each phase for biological material collection. Euthanasia was performed by electronarcosis followed by exsanguination. Tissue samples from the magnum (1 cm from the middle and thickest region per bird) and uterus (1 cm² from the middle region per bird) were collected from seven birds per treatment and age at the end of each phase. Until the pre-laying phase, samples were collected only from birds that had already started laying.

The samples were fixed in 10 % buffered formalin in phosphate-buffered saline (PBS, pH 7.2) for 24 h, following Junqueira and Junqueira (1983). Standard histological processing was conducted, and 5-µm-thick transverse sections were stained with Alcian Blue and Periodic Acid-Schiff (PAS) histochemical reaction. Samples were analyzed using an Olympus BX53F microscope (Tokyo, Japan) equipped with a digital camera (Olympus DP73). Digital images were acquired using the cellSens Dimension® software. For each analyzed variable, 10 measurements per section per bird were taken at 20x magnification, totaling 70 observations per variable per treatment.

The studied variables included fold height and width (µm) and the number of folds (units) in the magnum and uterus when birds were 17, 23, and 54 weeks old. Fold height was measured from the mucosa to the apical epithelium, considering only intact folds. Fold width was defined as the horizontal distance from the central height of the fold. The number of folds was determined randomly, counting only fully visible folds within the scanning field and excluding incomplete ones. A single evaluator conducted all histomorphometric analyses to avoid interpretation errors.

### Statistical analysis

Descriptive statistical analysis was used to evaluate production responses, with results presented as frequencies and percentages. Data were organized in tables and figures, considering multiple attributes and dimensions. Data analysis was performed using Microsoft Excel (Windows 2010), with means calculated using the program’s built-in tools.

Reproductive morphometry variables were analyzed using SAS (SAS Institute, 2011). Variance homogeneity was verified, followed by factorial model analysis, including the effects of treatments, sources, and levels of microminerals, as well as their interactions. The statistical model used was:

$$Y_{ijk}=\mu +F_{i}+N_{j}+{\left(F\times N\right)}_{ij}+e_{ijk}$$

where:

• ***Y_ijk_*** represents the response variable of the kkth experimental unit subjected to treatment (*i, j*);
• ***μ*** is the overall mean;
• ***F_i_*** corresponds to the effect of the iith mineral source;
• ***N_j_*** corresponds to the effect of the jjth mineral level;
• **(*F*×*N*)*_ij_*** represents the interaction between mineral source and level;
• ***e_ijk_*** is the experimental error, assumed to be normally distributed.

Means were compared using Tukey’s test at a 5 % significance level.

## Results and discussion

According to the data presented in Table 5, the initial performance of the birds showed a significant interaction between the sources and levels of Zn, Cu, and Mn supplementation on feed conversion from 1 to 3 weeks of age (FCR, 1-3 w) (*P* = 0.0326), as detailed in Table 5. However, no isolated effect (*P* > 0.05) of the sources, levels, or their interaction was observed on the other initial performance parameters, including initial weight, final weight, weight gain, feed intake, and feed conversion.

**Table 5:** Effect of supplementation with different sources and levels of zinc, copper, and manganese on the initial performance (1 to 15 weeks) of replacement pullets.

Effects	Variables	IW1d (g)	FW3w (g)	WG 1-3 w (g)	FI 1-3 w (g/bird/day)	FCR 1-3 w (kg/bird)	IW 4 w (g)	IW 6 w (g)	WG 4-6 w (g)	FI 4-6 w (g/bird/day)	FCR 4-6 w (kg/bird)
Sources	Sulfate	45.52	232.49	186.97	419.09	2.24	232.49	502.36	269.87	780.24	2.89
	Chelate	45.49	229.35	183.86	403.68	2.20	229.35	493.18	263.83	772.96	2.93
*P-value*		0.9434	0.1413	0.1383	0.9920	0.1304	0.1413	0.5141	0.7626	0.4533	0.4731
CV (%)		0.82	3.04	3.74	3.98	4.04	3.04	2.33	4.40	7.91	10.24
SEM		0.071	1.353	1.339	3.366	0.017	1.353	2.182	2.163	11.594	0.055
Levels	32/8/32	45.51	231.66	186.15	407.75	2.19	231.66	502.43	270.77	771.93	2.86
Zn/Cu/Mn	64/16/64	45.50	230.17	184.67	415.02	2.25	230.17	493.11	262.94	781.27	2.98
*P-value*		0.9812	0.1976	0.1934	0.2616	0.1054	0.1976	0.5072	0.8640	0.5927	0.5986
CV (%)		0.82	3.10	3.82	4.33	3.98	3.10	2.32	4.29	7.90	10.04
Sources x Levels											
*P-value*		1.0000	0.2382	0.2334	0.4203	0.0326	0.2382	0.9671	0.4359	0.4895	0.3631
Effects	Variables	IW 7 w (g)	FW 12 w (g)	WG 7-12 w (g)	FI 7-12 w (g/bird/day)	FCR 7-12 w (kg/bird)	IW 13 w (g)	FW 15 w (g)	WG 13-15 w (g)	FI 13-15 w (g/bird/day)	FCR 13-15 w (kg/bird)
Sources	Sulfate	502.36	1015.87	513.51	2393.69	4.67	1015.87	1193.46	177.59	1433.26	8.24
	Chelate	493.18	1019.02	525.84	2413.09	4.60	1019.02	1186.96	167.94	1457.31	8.82
*P-value*		0.5141	0.6201	0.3923	0.2725	0.7974	0.6201	0.6001	0.2995	0.2441	0.1227
CV (%)		2.33	2.98	4.83	2.97	5.63	2.98	2.04	14.99	5.35	14.31
SEM		2.192	5.730	4.744	13.490	0.049	5.730	4.589	4.894	14.613	0.231
Levels	32/8/32	502.43	1026.23	523.80	2428.82	4.64	1026.23	1196.18	169.94	1431.11	8.58
Zn/Cu/Mn	64/16/64	493.11	1008.66	515.55	2377.96	4.63	1008.66	1184.25	175.59	1459.46	8.48
*P-value*		0.5072	0.9403	0.8439	0.8003	0.9271	0.9403	0.4801	0.5697	0.2268	0.2659
CV (%)		2.32	2.85	4.92	2.79	5.69	2.85	2.00	15.17	5.32	14.72
Sources x Levels											
*P-value*		0.9671	0.6662	0.6259	0.3609	0.9665	0.6662	0.7479	0.4338	0.3347	0.2148
Sources x Levels						32/8/32 Zn/Cu/Mn				64/16/64 Zn/Cu/Mn	
						2,18B				2,30A	
						2,20				2,19	

Treatment means followed by different letters (in rows) differ significantly according to Tukey's test (5 %); CV (%): Coefficient of variation.

Through the interaction data, it is observed that FS+NR had the best feed conversion ratio (FCR) at 1-3 weeks, while increasing the supplementation levels of Zn, Cu, and Mn led to a higher FCR, resulting in the worst FCR among all treatments (FS+NS) for this age. This may have occurred due to an increase in feed intake (FI) and a reduction in weight gain (WG). On the other hand, treatments using FQ had similar average FCR values, even with increased supplementation levels, and did not differ statistically from each other.

According to Albino et al. (2014), birds undergo a multiphasic development, and from the 1st to the 6th week of age, visceral growth occurs. Additionally, since the intestinal mucosa undergoes continuous growth, it can be affected by both endogenous and exogenous factors (Maiorka et al., 2000). Based on these statements, it is believed that the gastrointestinal tract and its accessory glands were not yet sufficiently mature by the 3rd week of age to efficiently utilize the nutrients present in the FS+NS diet. Consequently, birds fed this diet consumed more feed than those fed the FQ diet (3.7 %) to meet their metabolic needs. Furthermore, due to the increased levels of Zn, Cu, and Mn, some antagonistic interactions may have occurred, leading to reduced weight gain (WG) and, consequently, affecting the feed conversion ratio (FCR). However, regardless of the source or supplementation level of the trace minerals, all reported variables were within the range recommended by the strain manual (Hy-Line, 2016) for this age.

When comparing the Zn, Cu, and Mn levels used in this study with those presented in the Brazilian tables for poultry and swine (Rostagno et al., 2017) for both inorganic and organic sources, it is evident that the highest level used in this study (64/16/64 ppm) is lower than the values recommended by the authors for inorganic mineral supplementation. On the other hand, the lowest level used in this study (32/8/32 ppm) is close to the values they suggest for using the organic source during the rearing phase. These results suggest that the levels recommended in the strain manual should be adopted for mineral supplementation using the inorganic source (sulfate) and that the recommendations in the Brazilian tables may be applied with slight variations when using the organic source (hydroxy analog of methionine).

The age at first egg for each treatment was determined based on the moment when the first egg was laid, which was considered as the attainment of sexual maturity. Birds fed the FS mineral diet, regardless of supplementation level, reached sexual maturity between 17 and 18 weeks of age, while birds in the FQ treatments reached maturity later (17 to 19 weeks). Thus, the age at first egg was 119 days (17 weeks) for birds receiving FS + NR trace minerals (T1), which also resulted in the highest egg weight (34.79 g) among the treatments in which first egg laying occurred in the same week (Table 6).

**Table 6:** Effect of supplementation with different sources and levels of zinc, copper, and manganese on sexual maturity (Age and egg weight) and onset of laying (Interval between the first and second egg) in commercial laying hens.

Mineral sources	Levels Zn/Cu/Mn	Age at first egg (days/weeks)*	First egg weight (g)	Light duration (natural+artificial)	Interval between the 1st and 2nd Egg (days/weeks)	Weight of the 2nd Egg (g)
Sulfate	T1 = 32/ 8 /32	119d. (17 w.)	34.79	13h00	25d. (21 w.)	50.92
	T2 = 64/ 16 /64	123d. (18 w.)	40.00	13h15	6d. (19 w.)	45.00
Chelate	T3 = 32/ 8 /32	131d. (19 w.)	42.13	13h30	1d. (19 w.)	44.29
	T4 = 64/ 16 /64	120d. (17 w.)	32.32	13h00	16d. (20 w.)	47.58

*Age at first egg was considered as a single egg and not the average of the first eggs from each replicate/treatment; **Age at second egg was considered as the second egg laid in the replicate where the first egg had been laid; however, the first and second eggs may have been from different hens, as the birds were not housed in individual cages.

According to Albino et al. (2014), the pre-laying phase is marked by constant physiological changes as the bird’s body prepares to begin egg production, increasing nutritional demands (especially for amino acids, calcium, and phosphorus). This is due to the completion of growth, the onset of reproductive system maturation, and the production of reproductive hormones. Thus, it is presumed that the primary reasons why FS outperformed FQ in terms of age at first egg are related to the birds' metabolism at this stage and the strength of the chelating agent binding to the trace minerals, making them unavailable due to potential antagonistic interactions with some nutrients at the intestinal lumen level.

Khatun et al. (2019) reported that the relative bioavailability and efficacy of organic trace minerals can vary depending on the chelating agent used, the binding strength, and the ligand-to-mineral ratio, potentially influencing their effectiveness. Inorganic sources, on the other hand, have high water solubility (Rijsselaere and Bruneel, 2018), whereas organic sources undergo less hydrolysis within the cell (Saldanha, 2008; Hartman, 2017). This may explain the increased utilization of inorganic Zn, Cu, and Mn, as they were more readily available. Additionally, zinc, copper, and manganese may have contributed to alleviating oxidative stress. It is important to emphasize that, regardless of the source, the function of minerals is not altered unless antagonistic reactions occur, preventing them from performing their roles.

According to Stevenson et al. (2019), metalloendocrinology states that hormonal function depends on minerals (calcium, zinc, copper, selenium, and iron) to interact at various stages of peptide hormone maturation.

A delay in the onset of laying was observed in commercial laying hens when using chelated minerals compared to inorganic minerals during the rearing phase (12 to 20 weeks), occurring at 139 days and 137 days with the chelated and inorganic sources, respectively (Santos, 2014). Our results corroborate theirs, as birds fed FS + NR of Zn, Cu, and Mn (T1) reached the age at first egg at 119 days.

Regarding the first egg weight, there was apparently no relationship between dietary supplementation with different sources and levels of Zn, Cu, and Mn sulfates and chelates, as there was a difference of up to three weeks between treatments in the onset of lay. According to Renema et al. (2007), the follicular development of pullets aged 18 to 22 weeks is closely related to feed intake and body weight. Thus, based on the texts of Renema et al. (2007), Stevenson et al. (2019) mentioned above, the first egg weight variable should not be influenced by Zn, Cu, and Mn supplementation, but perhaps the hormonal function on follicular development in pullets might be. This hypothesis needs further exploration to confirm its validity.

According to Hy-Line (2016), at 19 weeks of age, the expected egg weight is 42 g. Considering the same age, among the tested treatments, the first egg weight of birds in T3 (FQ + NR) already showed a similar weight, and from the second egg onward, it exceeded the reference manual value by 2.29 g.

Regarding the interval between the first eggs, it was observed that in treatments where the first egg was laid by the 17th week (T1 and T4), the interval between eggs was longer than in treatments where laying started from the 18th week of age (T2 and T3).

According to the strain manual (Hy-Line, 2016), this age marks the end of muscular system development and the beginning of fat cell production, characteristics that are relevant for laying hens. These physiological events may have influenced the birds until the end of the pre-laying phase (17 weeks) and affected the interval between eggs rather than the tested supplementation, as T1 (FS + NR) and T4 (FQ + NS) were supplemented with different sources and levels of Zn, Cu, and Mn, but had similar first egg weight and egg production interval.

Zuidhof et al. (2007) evaluated various indicators of reproductive and metabolic function in different strains and found an interval between sexual maturity and egg laying of 22.8 days, compared to 9.3 days in birds at 18 and 22 weeks, respectively. Hens that started laying at 18 weeks had eggs that were 3.6 g lighter compared to eggs from 22-week-old hens. Joseph et al. (2002) reported an increase of 2.4 g in egg weight following a delay in sexual maturity of three weeks (from 20 to 23 weeks of age). In the present study, the delay in sexual maturity was greater for birds fed the FQ + NR diet of Zn, Cu, and Mn (T3), resulting in a 4.97 g increase in egg weight when compared to the subsequent lay (20th week of age).

**Fig. 1:**
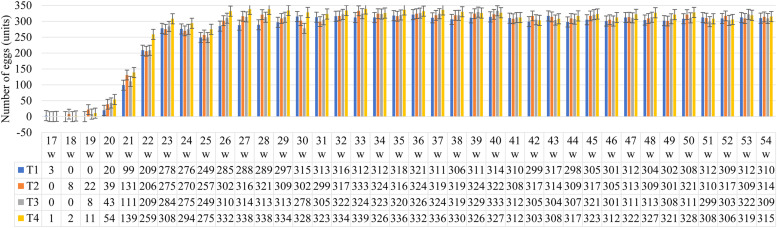
Effect of supplementation with different sources and levels of zinc, copper, and manganese on the number of eggs produced by light laying hens aged 17 to 54 weeks.

The number of eggs between 17 and 54 weeks of age in light laying hens is shown in Fig. 1. The number of eggs increased linearly from the 20th week, at which age birds from all treatments showed some production. However, between the 17th and 19th weeks of age, birds fed FS had a higher laying rate (33.3 %) compared to birds fed FQ. Regarding Zn, Cu, and Mn levels, NS resulted in a higher number of eggs for both sources (T2; T4) up to the 19th week. This result is attributed to the birds' metabolic needs during the pre-laying and early pre-peak phases, where FS may have been better absorbed under such conditions due to the birds' rapid growth rate. The presence of MHA-Ca in the diet may have contributed positively to this result.

As an alpha-keto acid of methionine, the calcium salt of the hydroxylated methionine analog (MHA-Ca) is primarily absorbed through diffusion (Nelson and Cox, 2014), and can therefore be used for protein synthesis like l-Met itself (Dibner and Knight, 1984), maintaining normal tissue concentrations of the nutrients in question.

Although the number of eggs increased at 20 weeks of age, it is important to highlight that, metabolically, at this age, birds of this strain were undergoing their third feather molt, their reproductive tract was still developing, and fat cell production was beginning (Hy-Line, 2016). However, puberty does not occur uniformly in birds (Albino et al., 2014).

During the pre-laying phase, hypothalamic maturity is a limiting factor for oviduct development and the formation of the first eggs. At this stage, birds undergo a complex physiological event due to interactions between the neuroendocrine and reproductive systems, as well as environmental factors, leading to responses associated with reproductive events and the manifestation of sexual behaviors (Morais et al., 2012b). According to Nys and Guyot (2011), the ovary, in interaction with the pituitary gland, controls all stages of ovum formation by secreting steroids and pituitary hormones. Albino et al. (2014) stated that hypothalamic maturity is more relevant than body weight for birds to reach sexual maturity, whereas D’Agostini et al. (2017) found a high correlation between the bird’s weight at six weeks of age and the age at which it reaches sexual maturity.

Trace minerals can influence the hypothalamic-pituitary-thyroid/adrenal axis depending on their concentration in the body (Henkin, 1976) or cause transient effects (Watts, 1990). The effects of trace minerals on endocrinology gained support from Pager's (1954) studies, which classified endocrine glands based on neurological control (sympathetic or parasympathetic) and the influence of minerals on neuroendocrine function. Another way minerals exert influence is by binding to hormone structures after secretion, either inhibiting or enabling biological activity (O’Halloran et al., 2013).

According to Kleyn (2013), a pullet has not yet completed its development when it starts laying, marking a new physiological state that requires dietary adjustments, including increased calcium levels for eggshell formation. Birds can store calcium for 14 days before laying their first egg, accumulating it in the medullary cavities of long bones (medullary bone), which is sufficient for producing six normal eggs (Kleyn, 2013).

Bouvarel et al. (2011) reported that each egg requires an average of 2.2 g of calcium, and when a pullet begins laying, estrogen release influences the availability of calcium from medullary bone. However, during this period, the birds’ individual nutritional requirements are higher than in the previous phase because, in addition to body growth (skeletal and muscular systems), the oviduct continues developing (Hy-Line, 2016), and the birds lack the physical capacity to exceed their habitual feed intake. Therefore, during sexual maturity establishment, birds must consume sufficient nutrients to support body growth, oviduct development, and ultimately, egg production (Kleyn, 2013).

Considering only the effect of the source, at 20 weeks, birds fed FQ produced more eggs than those fed FS. The levels showed a similar pattern regardless of the source, but birds receiving the NS level of Zn, Cu, and Mn laid more eggs (T2 and T4). It is assumed that as birds near the completion of reproductive tract development, FQ starts to outperform FS. Birds that retained more minerals for later absorption and higher trace mineral levels may have triggered a biological interaction in birds fed FS, either as an isolated or combined effect, given that its use was more efficient during periods of intense cellular, physiological, and enzymatic metabolism. Additionally, Zn, Cu, and Mn serve multiple functions, from forming simple enzyme structures to more complex processes such as DNA synthesis.

Birds fed FQ + NS (T4) produced the highest number of eggs between 20 and 23 weeks of age, with 16.7 % more eggs than the second-highest producing treatment (T2: FS+NS). This similar pattern across ages may be due to sexual maturity being reached, approaching peak lay. It is noteworthy that the avian reproductive system completes its development by 25 weeks of age (Hy-Line, 2016). A drop in egg numbers was observed in weeks 24 and 25, with reductions of up to 9.78 % (T1: FS + NR).

Summarizing, during the laying phase, the highest egg production occurred in different periods: 30 to 42 weeks (T1), 32 to 40 weeks (T2), 32 to 40 weeks (T3), and 26 to 40 weeks (T4). Based on these results, it is possible to conclude that FS concentrates its highest egg production between 30 and 42 weeks of age, and levels do not seem to have an effect at this stage. On the other hand, FQ levels appear to influence egg numbers, leading to an increase from NR to NS. Birds fed FQ + NS (T4) produced the highest number of eggs by the end of the study (10,743 eggs), whereas those fed T1 (FS + NR) produced the fewest (10,041 eggs).

Santos (2014), when testing the use of inorganic and organic trace minerals for commercial layers (12 to 80 weeks of age), found that an excess of available zinc in the diet improved feed energy utilization and resulted in greater fat deposition in birds, as the energy requirement for muscle building and growth had already decreased.

Zinc is associated with increased production, storage, and secretion of reproductive hormones (progesterone and estradiol) (Prasad et al., 1989). Additionally, zinc maintains normal vitamin A plasma concentration and is essential for the proper functioning of all ovarian epithelium (McDowell, 2003). This mineral has antagonistic interactions with calcium, phosphorus, copper, and iron (Silva and Pascoal, 2014).

Classic literature has identified copper as a promoter of productive traits due to its role in mechanisms affecting microbiota population dynamics and increasing serum mitogenic activity (Zhou et al., 1994), stimulating pituitary growth hormone secretion (Labella et al., 1973), and enhancing neuropeptide secretion (Barnea and Cho, 1987).

Manganese, like zinc, can negatively affect birds when present in high concentrations. Pine et al. (2005) observed endocrine system alterations impacting the production and secretion of sex hormones. According to Leeson and Summers (2001), manganese has low absorption efficiency and responds inversely to intake, meaning that increased Mn intake reduces absorption, and when absorption is high, intake decreases.

McDowell (1992) reported that manganese deficiency in laying hens resulted in reduced egg production and eggshell quality, lower hatchability, and an embryonic deficiency called chondrodystrophy.

**Fig. 2:**
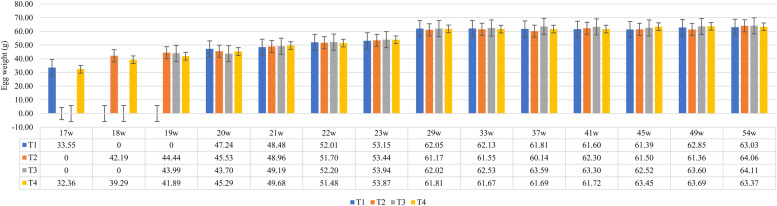
Effect of supplementation with different sources and levels of zinc, copper, and manganese on the egg weight of light laying hens aged 17 to 54 weeks.

Regarding egg weight, it followed the same pattern as egg production in relation to the sources and levels of the evaluated microminerals (Fig. 2). From the 17th to the 20th week, birds fed with FS in the diet produced slightly heavier eggs than those that received FQ, with the NR of Zn, Cu, and Mn resulting in the highest egg weight (47.24 g). From the 21st to the 54th week, the sources and levels showed very similar weights, without consistency to differentiate the influence of the observed treatments. However, FS had a higher average egg weight during the pre-lay and pre-peak phases, whereas in the laying phase, the average egg weight was higher for treatments with FQ. Nevertheless, this lower egg weight in birds that received FQ may be related to the lower bioavailability of the methionine source, as MHA-Ca has lower bioavailability compared to DL-Methionine.

The egg weights found in this study were predominantly higher than those described in the strain manual (Hy-Line, 2016).

Bibliographic reports indicate that egg weight is directly influenced by protein. Methionine is a precursor of protein synthesis, making it relevant for controlling egg weight, depending on the amino acid levels in the diet (Harms, 1999). Brumano et al. (2010) found that methionine+cystine levels positively influenced egg production, egg size, egg mass, and feed conversion per mass or dozen eggs in light layers. The calcium salt of the hydroxy analog of methionine is chemically different from DL and l-methionine, making it a methionine analog. Despite having lower metabolic activity in the calcium chelate source, it still has an absorption rate equal to or greater than DL-methionine (Zhang et al., 2015).

Zuidhof et al. (2007) reported that the maximum egg weight (52 g) is reached at 187 days (approximately 27 weeks), making it disadvantageous for laying to begin at 18 weeks, as it takes longer to reach the commercial egg weight. In the present study, hens from all treatments produced eggs weighing more than 52 g by the 23rd week of age, meeting one of the main current objectives in commercial layer production due to the economic importance of eggs (Savegnago et al., 2011).

**Fig. 3:**
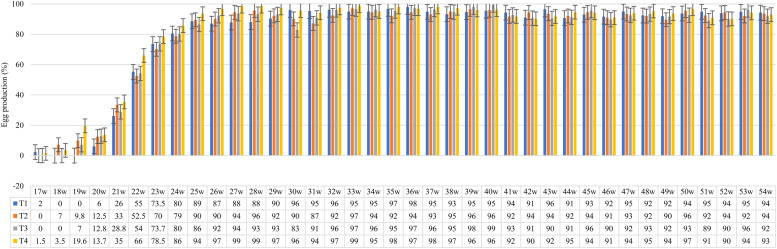
Effects of supplementation with different sources and levels of zinc, copper, and manganese on egg production of light laying hens aged 17 to 54 weeks.

It is known that egg number directly influences egg production (Fig. 3). The mineral sources and Zn, Cu, and Mn levels showed the best production index when hens were supplemented with FQ + NS (T4), while FS + NR (T1) and FQ + NR (T3) had the same egg production index per hen in the 23rd week. This is likely due to individual variations among the hens, such as pauses in egg production at different times, causing temporary fluctuations in production. In fact, the avian ovary is characterized by having multiple follicles at different maturation stages that are released consecutively. However, these sequences are marked by pauses that can last up to 40 h (Barbosa et al., 2013); this process is called follicular hierarchy.

The reproductive cycle of birds begins when the anterior pituitary starts producing gonadotropins, including follicle-stimulating hormone (which increases follicle size) and luteinizing hormone (which stimulates follicle ovulation and the development of interstitial cells responsible for producing sex hormones) (Freitas et al., 2011).

Referring to Pager (1954) information on the use of minerals in endocrinology, minerals are categorized as sympathetic, which can have stimulating/accelerating effects (Mn, P, Fe, Na, and Se) on the thyroid, anterior pituitary, adrenal medulla, and androgen-producing gonads, and parasympathetic, which can have sedative/decelerating effects (Zn, Cu, Ca, Mg, and Cr) on the pancreas, posterior pituitary, estrogen-producing gonads, parathyroid, and adrenal cortex, depending on their enzymatic and coenzymatic involvement.

Compiling the data from Pager (1954), Freitas et al. (2011), and the present study, it is assumed that among the evaluated microminerals, manganese is responsible for initiating egg production, as it acts as a stimulant on the anterior pituitary, the gland responsible for the main reproductive hormones (GH, FSH, and LH). The pituitary and pineal glands, which are influenced by photoperiod, have high concentrations of manganese (Leeson and Summers, 2001). According to Morais et al. (2012b), photoperiod is the main environmental factor regulating reproduction in birds.

Before ovulation, pre-ovulatory follicles have a region called the ovarian stigma, which has part of its surface composed of collagen. This is the site where follicular rupture occurs (Bahr and Johnson, 1991). Among the various functions attributed to the studied trace minerals, Zn, Cu, and Mn participate in collagen synthesis (Leeson and Summers, 2001). As follicular maturation progresses, collagenase activity increases, leading to the rupture of the stigma and the occurrence of ovulation (Nunes et al., 2013). In other words, Zn, Cu, and Mn may contribute to the release of the ovum, initiating egg formation in the infundibulum. According to Remena et al. (2007), the profile of sexual maturity can influence laying performance depending on the age at which it occurs. Birds from T4 (FQ + NS) had lower egg production in the early weeks (17 to 19 weeks) but showed a greater increase than the other treatments when they reached 25 weeks of age, possibly due to better uniformity of the birds within the group.

Based on the strain manual (Hy-Line, 2016), the expected egg production percentage in the pre-peak phase ranges from 9 to 89 %, and in the laying phase, from 93 to 94 %. When comparing different phases, similar results were observed between sources during the pre-peak phase, but NS outperformed the other levels, resulting in higher production percentages of 29 % (FS) and 33 % (FQ). In the laying phase, the treatments supplemented with FS achieved 93 % egg production, while those using FQ reached 94 %. The egg production observed in this study aligned with the values from the strain manual during the pre-peak and laying phases. The manual does not provide reference values for the pre-laying phase; therefore, no comparison was made for this period.

Santos (2014) supplemented organic and inorganic sources of trace minerals during the rearing phase (12 to 20 weeks) and concluded that the sources did not affect performance data during the laying phase (20 to 32 weeks). Min et al. (2018) studied the effect of increasing supplementation of zinc chelated to a methionine hydroxy analogue on laying hen performance and found a reduction in egg production in response to Zn-MHA addition. Li et al. (2018) observed a significant increase in laying rate, egg weight, egg mass, and improved feed conversion with 20 mg/kg of Mn, with no negative effect of the source type (organic or inorganic) on the reported parameters.

Regarding the morphological parameters of the magnum and uterus, no significant effect (*P* > 0.05) was observed for sources, levels, or their interactions concerning the height, width, and number of folds in the magnum and uterus of 17-week-old hens, as shown in Table 7.

**Table 7:** Effect of supplementation with different sources and levels of zinc, copper, and manganese on the magnum and uterus of light laying hens.

Effects	Variables	17 weeks of age					
		Magnum			Uterus		
		Fold Height (µm)	Fold Width (µm)	Number of Folds (Area)	Fold Height (µm)	Fold Width (µm)	Number of Folds (Area)
Sources	Sulfate	665.15	261.15	20.500	862.50	197.56	12.75
	Chelate	561.84	198.63	24.417	742.90	181.84	13.08
*P-value*		0.3951	0.9920	0.9341	0.2510	0.5600	0.5806
CV (%)		24.32	16.74	20.81	28.89	27.51	43.50
SEM		54.931	14.426	1.227	59.492	14.533	1.141
Levels Zn/Cu/Mn	32/8/32	614.41	234.74	22.08	847.70	197.23	10.33
	64/16/64	612.58	225.04	22.83	757.70	182.17	15.50
*P-value*		0.2143	0.4073	0.7569	0.2211	0.5550	0.9842
CV (%)		25.93	22.06	22.74	29.37	27.54	37.91
Source x Levels							
*P-value*		0.1897	0.3058	0.6719	0.1288	0.4253	0.5334
		23 weeks of age					
		Magnum	Uterus				
Effects	Variables	Fold Height (µm)	Fold Width (µm)	Number of Folds (Area)	Fold Height (µm)	Fold Width (µm)	Number of Folds (Area)
Sources	Sulfate	2724.40	858.34	2.1250	1878.00	232.10	6.83
	Chelate	2412.80	838.21	3.0417	1699.92	213.90	5.83
*P-value*		0.4622	0.6495	0.7267	0.6872	0.4425	0.2631
CV (%)		12.70	8.64	23.07	9.83	22.41	27.49
SEM		63.444	19.333	0.163	89.814	9.001	0.283
Levels	32/8/32	2631.57	848.22	2.50	1762.50	191.96	6.83
Zn/Cu/Mn	64/16/64	2505.60	848.34	2.67	1815.42	254.03	5.83
*P-value*		0.6830	0.7665	0.2675	0.2424	0.6413	0.2631
CV (%)		14.02	8.74	29.66	11.07	17.32	27.49
Source x Levels							
*P-value*	0.8470	0.7536	0.1827	0.2934	0.6036	0.1248	
	54 weeks of age						
		Magnum	Uterus				
Effects	Variables	Fold Height (µm)	Fold Width (µm)	Number of Folds (Area)	Fold Height (µm)	Fold Width (µm)	Number of Folds (Area)
Sources	Sulfate	2226.30	781.81	2.83	2295.20	237.95	6.17a
	Chelate	2111.10	595.41	3.50	1841.20	262.78	2.00b
*P-value*		0.5235	0.8506	0.3887	0.2440	0.6887	<0.001
CV (%)		16.24	25.38	36.68	16.43	13.57	25.25
SEM		71.516	34.709	0.232	58.231	6.774	0.199
Levels	32/8/32	2074.80	633.33	3.50	2007.79	247.70	4.16
Zn/Cu/Mn	64/16/64	2262.50	743.89	2.83	2128.54	253.03	4.00
*P-value*		0.2924	0.2224	0.8610	0.8384	0.9429	0.7096
CV (%)		15.82	27.93	36.68	19.95	14.52	60.07
Source x Levels							
*P-value*		0.0511	0.3722	0.5820	0.6644	0.9842	0.7682
Sources x Levels	32/8/32 Zn/Cu/Mn	64/16/64 Zn/Cu/Mn					
Sulfate		1933B	2519A				
Chelate		2216	2005				

A,B: Treatment means followed by different letters (in rows) differ significantly according to Tukey's test (5 %); CV (%): Coefficient of variation.

The data on height, width, and number of folds in the magnum and uterus of 23-week-old laying hens showed no effect of Zn, Cu, and Mn sources and levels on either tissue, nor any interactions between them (*P* > 0.05) (Table 7).

Table 7 presents the height, width, and number of folds in the magnum and uterus of 54-week-old laying hens.

There was an interaction (*P* = 0.0511) between Zn, Cu, and Mn sources and levels on the fold height of the magnum. However, no effect (*P* > 0.05) of sources and levels was observed on the height, width, and number of folds in the magnum at 54 weeks, nor any interactions (*P* > 0.05) for width and number of folds. A significant effect (*P* < 0.0001) of sources was found on the number of folds in the uterus, but no influence (*P* > 0.05) of levels or the interaction (*P* > 0.05) between sources and levels for this variable. Additionally, no significant effect (*P* > 0.05) of sources, levels, or their interactions was observed on uterine fold height and width.

There was an interaction between the supplementation levels of Zn, Cu, and Mn when the hens were fed FS, resulting in a greater fold height in the magnum when the birds were fed FS+NS. As supplementation increased, fold height also increased. No statistical difference was observed between sources or within the same supplementation levels. Table 7 presents the effects of the interaction between sources and supplemented levels on this variable.

These results may indicate an improvement in the efficiency of the egg formation process (specifically regarding the albumen), reducing the time the yolk and albumen remain in the magnum. This is because an increase in fold height suggests a greater number of albumin-secreting cells due to the epithelium becoming more extensive. Since this is the region of the oviduct responsible for protein synthesis, it is believed that the presence of MHA-Ca as a methionine source met the birds' nutritional requirements, contributing to higher productivity in the laying phase at 54 weeks, as observed in Fig. 3.

These statements are supported by the fact that this epithelium has mucus-producing characteristics. According to Wyburn et al. (1970), the presence of tubular glands in the magnum and well-developed folds increases the mucus secretion area. Methionine is the first limiting amino acid for laying hens and plays a crucial role in protein synthesis, organ development, and egg production and quality (Figueiredo Júnior et al., 2020).

Among the microminerals studied, it is not possible to determine which contributed most to the magnum's fold height. According to Butzen et al. (1985), the magnum is a region of the oviduct with the lowest Zn secretion in Japanese quail. In contrast, Rezende (2016) states that Zn is involved in albumen deposition, and Mabe et al. (2003) observed an increase in albumen weight with Cu supplementation.

The number of folds in the uterus at 54 weeks was higher with FS supplementation. Mineral supplementation with FS resulted in greater fold height and smaller width compared to FQ, leading to a higher number of folds per area in birds supplemented with FS+NR.

The morphological characteristics of the uterus in birds receiving FS supplementation likely resulted in a greater contact surface area, potentially enhancing eggshell formation and quality. These characteristics are considered beneficial, as they may help preserve the structures responsible for external egg quality, particularly the eggshell. According to King and McLelland (1979), the egg remains in the uterus for a variable period for calcium carbonate deposition, and morphological changes in the reproductive system may occur depending on the reproductive cycle phase (Morais et al., 2012a). Furthermore, eggshells become more fragile after 40 weeks of age due to an increase in egg size while maintaining a similar calcium content, leading to reduced shell thickness and quality (Furtado et al., 2001).

Zn is a cofactor of carbonic anhydrase, an enzyme essential for eggshell formation, and its deficiency reduces bicarbonate ion secretion (Leeson and Summers, 2001). Cu plays a role in forming eggshell membranes and the shell itself (Scheideler, 2008; Favero et al., 2013). Mn is responsible for activating enzymes involved in the synthesis of mucopolysaccharides, proteoglycans, and glycoproteins in the eggshell gland, contributing to the organic matrix formation in bones and eggshells (Georgievskii, 1982; Zhang et al., 2018).

Mabe et al. (2003) evaluated the effects of inorganic and organic sources of Zn, Cu, and Mn in corn- and soybean meal-based diets on egg quality. They found that Mn played a prominent role in eggshell formation, while Zn and Cu were mainly deposited in the yolk. The authors also reported that Zn and Cu could alter eggshell formation mechanisms due to their interaction with calcium carbonate. Zinc acts as an antagonist to calcium and copper (Silva and Pascoal, 2014). Additionally, Aguiar (2016) observed that different sources and levels of organic Zn (35, 50, and 65 mg/kg) and inorganic Cu (3.5 and 5 mg/kg) in Japanese quail diets resulted in increased reproductive epithelium height.

Histological results suggest that variations in the magnum and uterine epithelium may be associated with the birds' age, as ovarian function changes with aging until oviduct atrophy occurs. Consequently, reproductive hormone levels decline, egg production ceases, and nutrient absorption efficiency decreases in the gastrointestinal tract with advancing age.

Several studies support the relationship between zinc, copper, and manganese and hormone secretion. Zinc is involved in protein synthesis, gene expression regulation, and the storage, synthesis, and action of peptide hormones (Tapiero and Tew, 2003). Copper participates in LH synthesis and maintenance (Rajeswari and Swaminathan, 2014). Manganese plays a role in steroid hormone synthesis as it is involved in cholesterol metabolism (Xie et al., 2014), and its deficiency reduces reproductive hormone levels in laying hens (Wang et al., 2019).

**Fig. 4:**
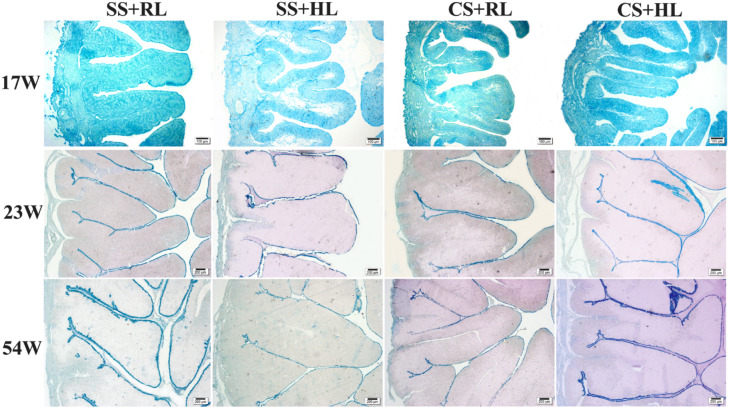
Photomicrographs of the magnum of laying hens at different stages: A - birds at 17 weeks of age, B - birds at 23 weeks of age, C - birds at 54 weeks of age, supplemented with different sources and levels of Zn, Cu, and Mn. Staining: Alcian Blue + PAS. Magnification: 20x. Scale bars: (A-B-C) 200 µm. Transversely sectioned.

**Fig. 5:**
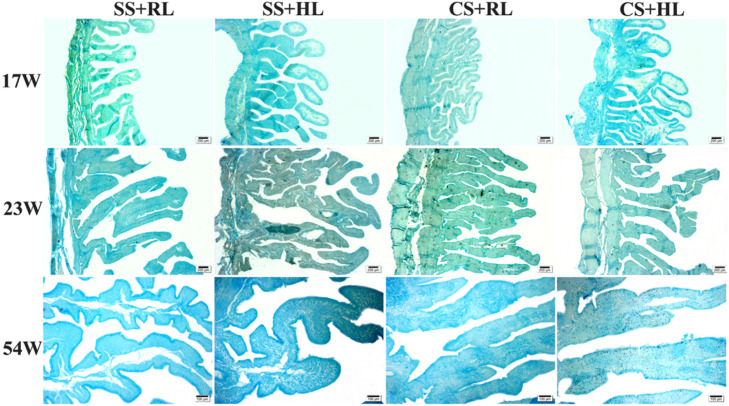
Photomicrographs of the uterus of laying hens at different stages: A – hens at 17 weeks of age, B – hens at 23 weeks of age, C – hens at 54 weeks of age supplemented with different sources and levels of Zn, Cu, and Mn. Staining: Alcian Blue + PAS. 20x. Scale bars: (A-B-C) 200 µm. Transversely sectioned.

The morphological aspects of the magnum and uterus in the evaluated treatments showed folds of similar sizes and shapes characteristic of each tissue, with differences depending on the oviduct region, bird age, and sexual maturity stage (Fig. 4, Fig. 5).

Upon reaching sexual maturity, mucosal modifications occur to enable albumen secretion (magnum) and successive eggshell layer formation (uterus or eggshell gland). Microscopic evaluation revealed differences in fold height, width, and number in the magnum and uterus due to supplementation with different sources and levels of Zn, Cu, and Mn at various ages.

Romanoff and Romanoff (1949) described the oviduct's growth as slow and progressive until 20 weeks, followed by rapid elongation around 21 weeks of age. In this study, with increasing age, magnum folds quadrupled in height across all treatments, while in the uterus, height increased at most twofold between 17 and 23 weeks of age. Consequently, the number of folds decreased by 87 % for both evaluated sources. At this stage, dynamic physiological changes occur to prepare the bird for egg production, and mineral supplementation likely contributed to anabolic processes, leading to the observed morphological expansions.

At 54 weeks, fold height and width were reduced, and the number of folds per area remained relatively unchanged. The reduction was less noticeable in birds receiving FQ+NS (T4), while FS+NR birds showed the most significant reductions (T1). Across all phases, the impact of increasing Zn, Cu, and Mn levels was unclear, but it is suggested that higher levels may reduce these variations.

According to Jeong et al. (2012), during egg formation, the oviduct tissues undergo extensive proliferation of luminal and glandular epithelial cells, extracellular matrix remodeling, and neovascularization. Meanwhile, Renema et al. (2007) emphasize differences in their study results due to variations in feed level and body weight of birds between 18 and 22 weeks, influencing ovarian development and follicle numbers. In this context, it can be reaffirmed that the modifications described in the magnum align with the literature and may have been influenced by the tested diet.

The magnum is a long component of the oviduct, with the presence of tubular glands and well-developed folds that allow the secretory area of the mucosa to increase threefold (Wyburn et al., 1970); in this region, there is more mucus than in other parts of the oviduct (Sisson and Grossman, 1986). On the other hand, the folds of the uterus are highly intersected by many transverse and oblique grooves, thus being subdivided into numerous tall, leaf-like lamellae (Sisson and Grossman, 1986). The use of different sources and levels of Zn, Cu, and Mn in the present study did not negatively affect the morphophysiology of laying hens during the development of the reproductive system in the pre-laying phase (17 weeks), pre-peak phase (23 weeks), and laying phase (54 weeks).

The uterus exhibited continuous growth from one phase to another, with FS promoting greater fold height and width at 17 and 23 weeks of age, while FQ showed higher mean width at 54 weeks. Regarding the levels, at 17 weeks, NR outperformed NS, but at 23 and 54 weeks, hens supplemented with NS had greater fold height and width than NR.

Schraer and Schraer (1965) reported that Zn was found in all segments of the oviduct, not just in the isthmus and uterus, with similar levels across all tissues. Cu concentration was higher in the isthmus and remained elevated in this tissue regardless of the developing egg's location. Mn was present in all oviduct segments, with fluctuations from ovulation to the egg's arrival in the uterus, suggesting that the magnum is responsible for transporting this metal into the egg, as a significant drop in manganese concentration occurs after it enters the shell gland.

According to Ball et al. (1999), the high Cu concentration in the isthmus may indicate a greater presence of copper-dependent metallothioneins, such as cuproenzyme and cuproprotein. Metallothioneins are the primary intracellular storage sites for copper and zinc ions (Cruz and Soares, 2011).

Pereira et al. (2020) supplemented laying hens with inorganic and organic sources of Zn, Mn, and Cu from the first day of life and concluded that the organic source promoted longer intestines and increased hormone secretion, leading to greater oviduct development during peak production.

Aguiar (2016) evaluated the effect of an organic zinc source and an inorganic copper source in 180-day-old Japanese quails and found that these microminerals indirectly influenced increased cell synthesis, reflected in greater magnum epithelial height and mucin production.

The microscopic morphology of the magnum and uterus in laying hens demonstrates how distinct the physiological responses of birds can be depending on their diet, particularly when fed diets containing different sources and levels of microminerals. Further research and publications on this nutrient are necessary to enhance understanding, improve production, and promote animal welfare.

## Conclusions

The use of the sulfate source with the recommended levels of zinc, copper, and manganese (32/8/32 ppm) resulted in the best initial performance, age at first egg, and beneficial effects on the reproductive system of laying hens. However, further investigations are required to evaluate the long-term impact of these mineral sources on egg quality, hen health, and overall productive performance throughout the laying period.

## Disclosures

The authors declare that they have no other conflicts of interest.
